# Spatial Distribution and Habitat Overlap of Five *Columbidae* Species in the Czech Republic

**DOI:** 10.3390/ani12060743

**Published:** 2022-03-16

**Authors:** Kristina Floigl, Yanina Benedetti, Jiří Reif, Federico Morelli

**Affiliations:** 1 Faculty of Environmental Sciences, Czech University of Life Sciences Prague, Kamýcká 129, Suchdol, 165 00 Prague, Czech Republic; ybenedetti73@gmail.com (Y.B.); fmorellius@gmail.com (F.M.); 2 Institute for Environmental Studies, Faculty of Science, Charles University in Prague, Benatska 2, Prague 2, 128 01 Prague, Czech Republic; jirireif@natur.cuni.cz; 3 Department of Zoology and Laboratory of Ornithology, Faculty of Science, Palacky University Olomouc, 17. listopadu 50, 771 46 Olomouc, Czech Republic; 4 Czech Society for Ornithology, Na Bělidle 34, Praha 5, 150 00 Prague, Czech Republic

**Keywords:** *Columbidae*, Czech Republic, land use composition, habitat overlap, species distribution

## Abstract

**Simple Summary:**

The spatial distribution of species and their utilisation of resources are essential for better understanding species ecology. Using data gathered by ornithologists in Czech Republic, we studied land use type utilisation of five pigeons and doves. Our study aimed to understand which species and type of land use are positively associated and whether the species are positively associated with land use heterogeneity. Additionally, we quantified the amount of land use type utilisation by each species and their spatial overlap in these land use types. We hypothesised that the species would mostly overlap in farmlands and urban areas. We found an almost complete overlap between the domestic pigeon (*Columba livia domestica)* and the Eurasian collared dove (*Streptopelia decaocto*), as well as between the common wood pigeon (*Columba palumbus*) and the European turtle dove (*Streptopelia turtur*). We confirmed our hypothesis that the species distribution not only overlapped in farmlands and urban areas, but also in forests. Our study provides insight into these common species distributions and habitat affinities.

**Abstract:**

Habitat overlap occurs when two species co-exist in the same habitat and utilise the same resources. Using common bird monitoring data in Czech Republic from 2015 and 2016, we compared the affinities of five *Columbidae* species regarding land use types. Moreover, we analysed the effects of land use types and land use heterogeneity on five species distributions. The aim of the study was to quantify the habitat overlap of five *Columbidae* species regarding types of land use and land use heterogeneity. We predicted a high level of habitat overlap between most of the species and its occurrence in farmlands and urban areas. Our results confirmed the high habitat overlap of all five *Columbidae* species in farmlands. An almost complete overlap was recorded between *Columba livia domestica* and *Streptopelia decaocto*, as well as between *Columba palumbus* and *Streptopelia turtur*. Considering land use utilisation, *C. livia* and *S. decaocto* mainly utilised farmlands and urban areas. Furthermore, deciduous forests were utilised by *Columba oenas* and coniferous and mixed forests by *C. palumbus*. Finally, *S. turtur* mainly utilised grasslands and avoided urban areas. We conclude that *Columbidae* species overlap in spatial distributions, mostly in urban areas, forests, and farmlands. Our study provides a summary of these common species habitat affinities.

## 1. Introduction

Species ecological niche is a complex concept that reflects relationships to the environment, which are developed throughout the evolution of each species [1,2,3]. By adapting morphological, ecological, and physiological characters, species occupy ecological niches defined by environmental conditions and available resources. When two species co-exist in the same habitat, the habitat overlap occurs [4]. Under the conditions of habitat overlap, species compete for the common resources and this competition ultimately results in the local extinction of a less effective competitor [5] and/or in niche partitioning [6]. This process allows competing species to utilise the same resources in different ways, and thus promotes the co-existence of species [7].

Habitat loss may contribute to the increased habitat overlap in European landscapes due to agricultural practices and deforestation [8,9]. To avoid this overlap, species are colonising other suitable and available habitats, such as urban areas [10]. Colonisation of urban areas by birds has been occurring since the middle of the 20th century, and it is a process consisting of three stages: Arrival, adjustment, and spread [11]. At the same time, closely related species require a long time to develop interspecific differences [12]. Moreover, the number of species that have adapted to cities is still low since a small amount of species have developed the ecological and life history traits that lead them to be urban-tolerant species [13]. As a result, low interspecific competition and low species richness occur in cities, which are accompanied with high habitat overlap in urban areas.

The members of *Columbidae* family rank among the most common and widely distributed species in European landscapes. Their distribution overlaps in habitats, such as farmlands, urban areas, and natural forests [9]. To date, previous studies have analysed diet [14] and nest site overlap [15] in the *Columbidae* family. Additionally, several studies on *Streptopelia turtur* (Linnaeus, 1758) in the Mediterranean region focus on the effects of different types of landscape on the species [2,3,4,5,6,7,8,9,10,11,12,13,14,15,16]. However, to our knowledge, there are no records of studies concerning the distribution and habitat overlap of *Columbidae* species breeding in the Czech Republic, i.e., feral pigeon (*Columba livia forma domestica* Linnaeus, 1766), stock dove (*Columba oenas* Linnaeus, 1758), wood pigeon (*Columba palumbus* Linnaeus, 1758), Eurasian collared dove (*Streptopelia decaocto* Frivaldszky, 1838), and European turtle dove (*Streptopelia turtur*). According to a study on the performance of molecular phylogeny, *Columba* and *Streptopelia* species belong to different clades, i.e., *Columba* belong to the Old World clade and *Streptopelia* to a separate clade [17]. These birds may be an interesting group for studying habitat overlap due to the recent colonization of urban areas by some of these species [18,19]. For instance, in Finland, researchers have reported an increase in the abundance of *C. palumbus* in cities [20]. Furthermore, *C. palumbus* and *S. decaocto* are recognised as urban species and their populations are increasing in urban areas in Britain [21] and Baltic region [22]. In addition, according to specialisation traits in species tolerant to urbanisation, *C. livia* has the second highest relative urban tolerance score [13]. From the case of the Czech Republic, *S. turtur* is not considered an urban species due to its feeding behaviour, which requires open habitats, such as farmlands and grasslands [23]. *S. decaocto* is almost exclusively an urban species, and *C. palumbus* colonised urban areas recently in comparison to other European countries, where the colonisation process occurred earlier in the century [11].

Quantifying habitat overlap provides insight into species competition in specific habitats, thus allowing us to understand in which habitats competition is occurring and which types of land use require more conservation attention [2]. Here, we specifically focus on the role of landscape heterogeneity in the association with *Columbidae* species since it can provide more insight into species habitat type affinities. For instance, whether they are attracted to open space habitats with lower edge density, such as farmlands or to habitats with high edge density and fragments, such as urban areas. For this purpose, our study assesses whether two landscape heterogeneity metrics, land use richness and edge density, are significant predictors of *Columbidae* species distribution.

Moreover, studying the ecology of species, such as *C. oenas* and *S. turtur*, can provide insight for conservation strategies due to their potential to serve as umbrella species. Commonly, effective umbrella species should cover a large geographical area, and have high spatial and habitat overlap with co-occurring species [24,25]. For instance, *S. turtur* is a vulnerable species listed on the Annex II of the Birds Directive, that aims to allow for the hunting of species to be sustainable [26]. Therefore, the prohibition of hunting *S. turtur* during specific periods allows other farmland species to benefit from these measures, as well.

The main aim of our study is to quantify the habitat overlap of five species of the *Columbidae* family present in the Czech Republic regarding land use composition and land use heterogeneity. Within this goal, our objectives were to map the species, calculate the species habitat overlap index, assess the habitat selection, and determine the relationship of land use types and landscape heterogeneity with the occurrence of the following five species: *C. livia*, *C. oenas*, *C. palumbus*, *S. decaocto*, and *S. turtur*. We predicted a high level of habitat overlap among most of the species, and that the habitat overlap would occur in farmlands and urban areas [11] due to the species spatial distribution and the detected increasing number of *Columbidae* species in urban areas. Furthermore, regarding species phylogeny, we can predict two possible outcomes: (i) Species within the same clade will show a greater overlap than distantly related species from different clades due to the relatively short time since their divergence; (ii) closely related species from the same clade will show a smaller overlap due to the higher interspecific competition.

## 2. Materials and Methods

The data were collected at 118 study sites scattered in different land use types and altitudes throughout the Czech Republic within the Bird Breeding Monitoring Program in 2015 and 2016. The Program is conducted by voluntary ornithologists using point counts [27]. Each site is represented by a transect comprising approx. 20 sampling points located 300–500 m apart. In total, 2324 point counts were visited. Birds are visited twice per breeding season at each sampling point, to cover both early and late breeders. During one visit, birds detected both visually and acoustically are counted for 5 min at each sampling point. We considered only the counts within a 100-m radius around each sampling point.

**Figure 1:**
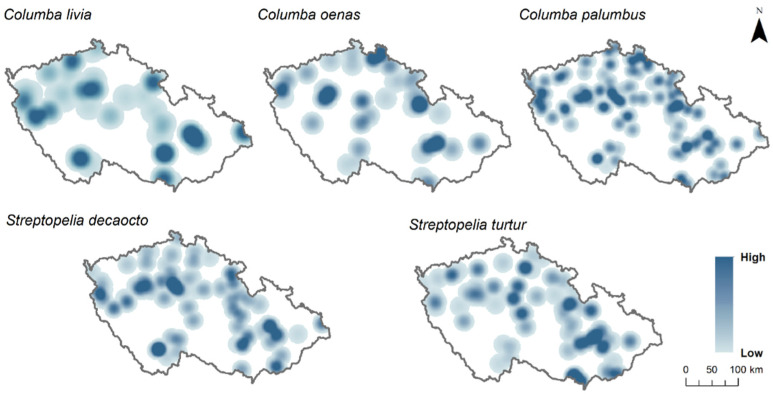
Five Columbidae species distribution in the Czech Republic with Kernel density interpolation. The darker blue colour represents a high density of points, where species were present during the survey; light blue colour represents a low density of points, where species were present during the survey; white areas are where census transects are missing. Distribution of five species: Feral pigeon (*Columba livia forma domestica*), stock dove (*Columba oenas*), wood pigeon (*Columba palumbus*), Eurasian collared dove (*Streptopelia decaocto*), and European turtle dove (*Streptopelia turtur*).

**Figure 2:**
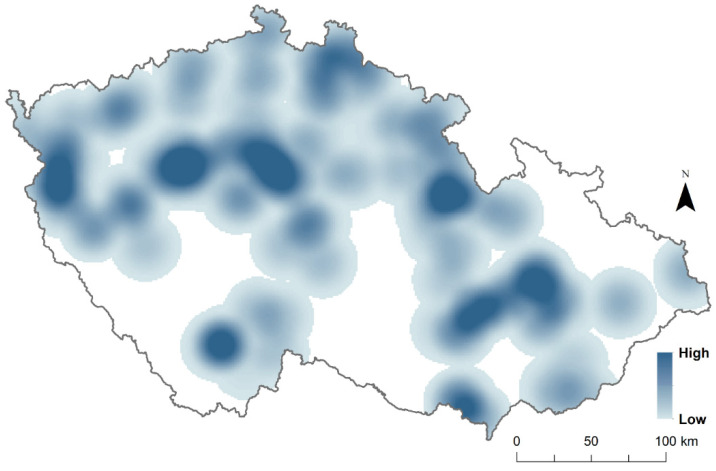
Species richness distribution of five *Columbidae* species in the Czech Republic with Kernel density interpolation. The darker blue colour represents higher species richness; light blue colour represents lower species richness; white areas are where census transects are missing. The included species are feral pigeon (*Columba livia forma domestica*), stock dove (*Columba oenas*), wood pigeon (*Columba palumbus*), Eurasian collared dove (*Streptopelia decaocto*), and European turtle dove (*Streptopelia turtur*).

In this study, we used the presence and absence of four Columbidae species (specifically, *C. livia*, *C. oenas*, *C. palumbus*, *S. decaocto*, and *S. turtur*) at the sampling points. Presence at sampling points was attributed when the species was observed at least once during the 2 years of observations, while absence was assumed when the species was absent in both survey years. For better visualisation of sampling points where each species was present and species richness per each sampling point, we mapped the sampling points using the Kernel density interpolation method from ArcGIS (Figure 1 and Figure 2).

The land use map of the study area was provided by the Nature Conservation Agency of the Czech Republic as the consolidated layer of ecosystems (CLE). CLE is based on a country-wide habitat mapping performed during the early 2000s and updated to 2018 [28]. We used ESRI 2011. ArcGIS Desktop: Release 10. Redlands, CA, USA: Environmental Systems Research Institute [29] to calculate land use composition around the 100-m radius of each point count. Land use composition consists of seven land use types: Deciduous, coniferous, and mixed forests, farmlands, grasslands, urban areas, and land use types, which were grouped into one category as “other”, such as shrubs, quarries, rocks, and water. Furthermore, to quantify landscape heterogeneity, we calculated two landscape metrics: Land use richness, which is the number of different land use types per site [30], and edge density, which is the ratio between total lines or perimeters and total area of each site [31]. Both landscape metrics were calculated within the 100-m radius of each point count.

We compared the Columbidae species affinities regarding the land use types using the function “habitat overlap”, from the “indicspecies” package in RStudio, which compares pairs of resource niches. The function returns the overlap index between each pair of species [32], using the amount of resource utilised by each species. The index value ranges from zero (no resources are shared by the two species) to one (all of the resources are shared).

To analyse the differences in utilisation of land use among the species, we used the habitat overlap module from the “EcoSimR” package in RStudio [33]. It allowed us to plot the resource utilisation matrix. The plot provides a visualisation of the observed utilisation matrix, the area of each circle is proportional to the utilisation of a land use type by each species. If there is no circle, the utilisation matrix is zero.

To analyse the effects of land use types, land use richness, and edge density on species distribution, we performed generalised linear models (GLM) [34] for each species. To account for the potential spatial autocorrelation (SAC) between sampling points, we applied a Mantel test [35]. The Mantel statistic (r_M_) varies between −1 and +1. It evaluates the similarity between two matrices, first calculated as a geometric distance, and the second one with a geographical distance among the sampling sites [36]. To test for the significance on the Mantel test, we ran the Monte Carlo permutations with 999 randomisations [37]. No significant autocorrelation was detected in the dataset used for the analyses (Mantel test, 999 randomisations: r_M_ = 0.09, simulated *p* > 0.05). Furthermore, to assess the relationship between species richness and land use types, as well as between land use richness and edge density, we performed a separate GLM with Poisson distribution.

In the GLM species, presence and absence were used as a response variable assuming a binomial distribution. In addition, land use type (deciduous forest, coniferous forest, mixed forest, urban areas, farmlands, grasslands, etc.), land use richness, and edge density, were used as predictors. After building full models, i.e., the models containing a complete set of predictors, for each species, a test of variance inflation factor (VIF) was applied to check for potential multicollinearity issues among predictor variables, using the function “check_collinearity” from a “performance” package [38] for RStudio. Only variables with VIF < 6 were introduced in the final models [39].

All of the statistical tests were performed with RStudio: Integrated Development for R. RStudio, PBC: Boston, MA, USA [40].

## 3. Results

The habitat overlap analysis included a total of 2324 sampling points of presence and absence of five Columbidae species, seven different land use types, and two landscape metrics. The most widely distributed species of Columbidae in the Czech Republic was *C. palumbus*, with 62% (*n* = 1448) of occupied points, followed by *S. decaocto* with 21% (*n* = 499), *S. turtur* with 15% (*n* = 357), *C. oenas* with 8% (*n* = 191), and *C. livia* with 7% (*n* = 168) of the total occurrence in the country (Figure 1). In addition, no sampling point had all five species present, nine sampling points had four species present, 131 sampling points had three species present, 558 sampling points had two species present, and 1118 sampling points had one single species present (Figure 2).

**Figure 3:**
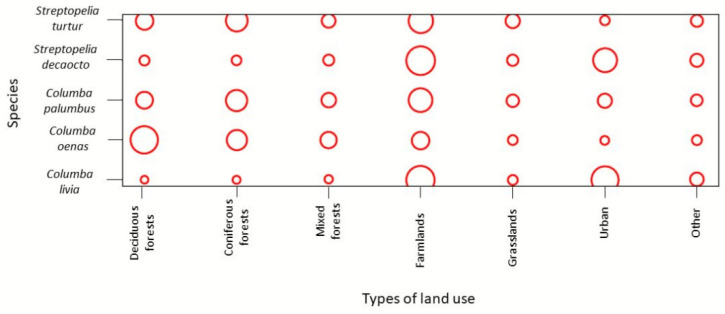
Habitat utilisation matrix based on land use composition around sampling points of five species from the family Columbidae in the Czech Republic. The figure provides a graphical portrayal of the observed utilisation matrix, and the increasing area of each circle is proportional to the increasing utilisation of each land use type by each species. If no circle is shown, the utilisation is zero. There are seven types of land use.

The habitat overlap index based on land use composition at sampling points shows an almost complete habitat overlap between pairs of *C. livia* and *S. decaocto*, as well as between *C. palumbus* and *S. turtur*. On the contrary, the smallest habitat overlap was estimated between *C. livia* and *C. oenas*, as well as between *S. decaocto* and *C. oenas*. Furthermore, *C. oenas* had an 0.8 habitat overlap index with *S. turtur* and *C. palumbus* (Table 1). When looking at the mean habitat overlap, *C. palumbus* had the highest habitat overlap with the other Columbidae species, followed by *S. turtur*. On the contrary, *C. oenas* had the lowest mean habitat overlap (Table 1). According to the utilisation plot, mixed forests, farmlands, grasslands, and other land use types were equally used by all five species, except for *C. oenas*, which utilised farmlands less than the other species. In deciduous forests, mostly *C. oenas* was present, while in coniferous forests, in addition to *C. oenas*, *C. palumbus* and *S. turtur* were present. Urban areas were inhabited mainly by *C. livia* and *S. decaocto* (Figure 3).

The presence of *C. livia* was negatively associated with deciduous, coniferous, and mixed forests, farmlands, grasslands, and other types of land use, and positively with edge density (Table 2). *C. oenas* showed negative associations with farmlands and urban areas, and positive associations with deciduous and mixed forests (Table 3). A model for *C. palumbus* showed only the positive associations—with coniferous and mixed forests (Table 4). *S. decaocto* was negatively associated with deciduous, coniferous, and mixed forests, farmlands, grasslands, and other land use types. However, a positive association was observed with edge density in this species (Table 5). *S. turtur* was negatively associated with urban areas and edge density. However, it showed a positive association with grasslands (Table 6).

Species richness was positively associated with urban areas and edge density. However, coniferous forests and land use richness showed a negative association with species richness (Table 7).

## 4. Discussion

Our results confirmed the hypothesis of high habitat overlap among all five *Columbidae* species in farmland areas. However, they mainly co-exist in three land use types: Farmlands, forests, and urban areas. Furthermore, we recorded an almost complete habitat overlap between *C. livia* and *S. decaocto*, as well as between *C. palumbus* and *S. turtur*. Regarding land use utilisation, *C. livia* and *S. decaocto* mainly utilised the farmlands and urban areas, and avoided all three types of forests (i.e., deciduous, coniferous, and mixed forests), grasslands, and other land use types. At the same time, forests were utilised mainly by *C. oenas* and *C. palumbus*. Specifically, *C. oenas* mainly utilised deciduous forests and avoided the farmlands and urban areas. *C. palumbus* utilised coniferous and mixed forests. Finally, *S. turtur* utilised mostly grasslands and avoided urban areas. Regarding species richness, it was higher in urban areas and areas with higher edge density. However, it was lower in coniferous forests and areas with higher land use richness.

Almost all of the complete habitat overlap between *C. livia* and *S. decaocto* was associated with their presence in areas with higher edge density, i.e., in urban areas. Therefore, habitat overlap between these two species is not surprising since both species are common in urban avian assemblages [21]. Additionally, high habitat overlap was recorded between *C. palumbus* and *S. turtur*, mainly in farmlands, deciduous, and coniferous forests. The overlap of these species in farmlands occurred due to the fact that *C. palumbus*, which used to be a forest specialist, started colonising other habitats, such as farmlands in the 21st century in the Czech Republic [41]. In addition, *S. turtur* is a highly specialised species that has specific foraging habitat [42], which depends on open farmlands and grasslands [23]. Furthermore, *C. palumbus* was positively associated with forest, specifically coniferous and mixed stands, probably due to the fact that it depends on the shrubs and trees as nesting habitats [43]. However, several studies in western and northern Europe have reported that *C. palumbus* colonised urban areas in the beginning of the 21st century [20,21,22]. Although our results did not show a positive association between *C. palumbus* and urban areas, the species was present in cities. The lack of positive association in our study could be due to the fact that the colonisation process is still in progress since it started later in the eastern parts of Europe [11], including the Czech Republic or due to the sample bias and lack of sampling points in urban areas.

Regarding *C. oenas*, it had the lowest mean habitat overlap with all of the species. Therefore, it was the least congruent with other species distributions. However, the high habitat overlap occurred with *C. palumbus* mainly in forests, which aligns with the aforementioned results since both species utilise this land use type. Moreover, *C. oenas* was primarily present in deciduous and mixed forests since it depends on old beech trees for nesting [41]. Consequently, we can say that *C. oenas* is a forest specialist, confirmed by previous studies from the Czech Republic that have listed it as a forest species [44,45]. Furthermore, *C. oenas* showed a negative relationship with urban areas, which is in accord with its aforementioned preference for old trees [41], which are not common in cities. Regarding habitat overlap, *C. oenas* overlapped with *S. turtur*, the second highest habitat overlap from all of the other species, which is not surprising since both species feed on farmlands. According to a new study, populations of farmland specialists, including *S. turtur*, have declined in Europe since the 1980s [46]. Therefore, exploring the possibility of using *S. turtur* as an umbrella species is useful for future conservation measures.

In general, landscape heterogeneity did not play an essential factor in the spatial distribution of the studied species. The results show that land use richness does not seem to impact the presence or absence of any of the studied species. However, edge density was an important predictor for three species. *C. livia* and *S. decaocto* are primarily present in urban areas where land use richness is low and edge density is high due to small fragmented patches [47]. Next, *S. turtur* is a farmland specialist, and thus requires open habitats, such as farmlands, for feeding. In addition, it is negatively associated with edge density.

The higher species richness present in urban areas and areas with higher edge density was expected since most of the studied species are associated with cities, except for *S. turtur*. On the contrary, species richness was lower in coniferous forests and in more heterogeneous areas, in terms of land use richness.

Interestingly, no sampling point had all of the five species present, and only nine sampling points had four species present. From these patterns, we can speculate that an interspecific competition exists between these five species of *Columbidae*. Furthermore, our predictions that closely related species, pigeons, will have lower habitat overlap than distantly related species, pigeons and doves, were confirmed with several cases. Habitat overlap was the highest between *C. livia* and *S. decaocto*; *C. palumbus* and *S. turtur*; and finally, between *C. palumbus* and *S. decaocto*. However, additional detailed studies are necessary to further confirm this hypothesis.

## 5. Conclusions

In conclusion, our results showed that the *Columbidae* species mainly co-exist in three land use types: Urban areas, forests, and farmlands. With the highest species richness in urban areas and areas with high edge density, this indicates that species have high tolerance to anthropogenic disturbances in cities. *C. livia* and *S. decaocto* had high habitat overlap in urban areas; *C. oenas* and *C. palumbus* had high habitat overlap in forests; and *C. palumbus* and *S. turtur* had high habitat overlap in farmlands. Furthermore, our research assumes that *S. turtur* can serve as an umbrella species for lowering the hunting pressure on other farmland and grassland species. Finally, our results provide a comprehensive insight into these common species habitat affinities and utilisation. Further research is necessary to understand the reasons behind drivers of their co-existence in these habitats.

## Figures and Tables

**Table 1:** Habitat overlap among five *Columbidae* species (feral pigeon (*Columba livia forma domestica*), stock dove (*Columba oenas*), wood pigeon (*Columba palumbus*), Eurasian collared dove (*Streptopelia decaocto*), and European turtle dove (*Streptopelia turtur*)) was estimated as a similarity index based on land use composition and two landscape metrics at each site where the species were present. Value of one suggests that the species are completely sharing resources. Value of zero indicates that the species do not share any resources. Mean—mean habitat overlaps for each *Columbidae* species.

	*C. livia*	*C. oenas*	*C. palumbus*	*S. decaocto*	*S. turtur*	Mean
*C. livia*	1					0.659
*C. oenas*	0.391	1				0.627
*C. palumbus*	0.735	0.820	1			0.838
*S. decaocto*	0.988	0.472	0.808	1		0.755
*S. turtur*	0.664	0.825	0.990	0.752	1	0.799

**Table 2:** GLM model results accounting for the presence and absence of species feral pigeon (*Columba livia forma domestica*) concerning different land use types, land use richness, and edge density in 2016. Abbreviations: SE—standard error. Significant variables are highlighted in bold.

	Estimate	SE	z-Value	*p*-Value
**(Intercept)**	**−1.136**	**0.393**	**−2.891**	**0.004**
**Deciduous forest**	**−0.042**	**0.008**	**−4.927**	**<0.001**
**Coniferous forest**	**−0.043**	**0.008**	**−5.708**	**<0.001**
**Mixed forest**	**−0.030**	**0.008**	**−3.971**	**<0.001**
**Farmland**	**−0.013**	**0.004**	**−3.398**	**0.001**
**Grassland**	**−0.024**	**0.006**	**−3.713**	**<0.001**
Urban	0.004	0.004	1.227	0.220
**Other land use types**	**−0.010**	**0.005**	**−1.847**	**0.065**
Land use richness	−0.051	0.063	−0.803	0.422
**Edge density**	**3.771**	**1.627**	**2.318**	**0.020**

**Table 3:** GLM model results accounting for the presence and absence of species stock dove (*Columba oenas*) concerning different land use types, land use richness, and edge density in 2016. Abbreviations: SE—standard error. Significant variables are highlighted in bold.

	Estimate	SE	z-Value	*p*-Value
**(Intercept)**	**−2.247**	**0.251**	**−8.962**	**<0.001**
**Deciduous forest**	**0.020**	**0.003**	**7.328**	**<0.001**
**Mixed forest**	**0.007**	**0.004**	**2.081**	**0.037**
Grassland	−0.007	0.005	−1.248	0.212
**Farmland**	**−0.007**	**0.003**	**−2.412**	**0.016**
**Urban**	**−0.013**	**0.006**	**−2.129**	**0.033**
Other	−0.002	0.006	−0.422	0.673
Land use richness	−0.005	0.073	−0.065	0.948
Edge density	−5.300	3.507	−1.511	0.131

**Table 4:** GLM model results accounting for the presence and absence of species wood pigeon (*Columba palumbus*) concerning different land use types, land use richness, and edge density in 2016. Abbreviations: SE—standard error. Significant variables are highlighted in bold.

	Estimate	SE	z-Value	*p*-Value
(Intercept)	0.245	0.291	0.842	0.4
Deciduous forest	0.002	0.003	0.745	0.456
**Coniferous forest**	**0.008**	**0.003**	**2.595**	**0.009**
**Mixed forest**	**0.007**	**0.003**	**2.081**	**0.037**
Farmland	−0.001	0.003	−0.354	0.723
Grassland	0.000	0.003	0.053	0.958
Urban	−0.004	0.003	−1.384	0.166
Other land use types	−0.002	0.004	−0.431	0.666
Land use richness	−0.005	0.034	−0.161	0.872
Edge density	1.600	1.324	1.209	0.227

**Table 5:** GLM model results accounting for the presence and absence of species Eurasian collared dove (*Streptopelia decaocto*) concerning different land use types, land use richness, and edge density in 2016. Abbreviations: SE—standard error. Significant variables are highlighted in bold.

	Estimate	SE	z-Value	*p*-Value
(Intercept)	0.127	0.345	0.368	0.713
**Deciduous forest**	**−0.038**	**0.005**	**−8.412**	**<0.001**
**Coniferous forest**	**−0.044**	**0.005**	**−9.631**	**<0.001**
**Mixed forest**	**−0.027**	**0.004**	**−6.185**	**<0.001**
**Farmland**	**−0.015**	**0.003**	**−4.849**	**<0.001**
**Grassland**	**−0.026**	**0.004**	**−6.010**	**<0.001**
Urban	0.003	0.003	1.005	0.315
**Other land use types**	**−0.019**	**0.004**	**−4.376**	**<0.001**
Land use richness	−0.005	0.048	−0.113	0.910
**Edge density**	**7.805**	**1.966**	**3.970**	**<0.001**

**Table 6:** GLM model results accounting for the presence and absence of species European turtle dove (*Streptopelia turtur*) concerning different land use types, land use richness, and edge density in 2016. Abbreviations: SE—standard error. Significant variables are highlighted in bold.

	Estimate	SE	z-Value	*p*-Value
**(Intercept)**	**−0.995**	**0.175**	**−5.688**	**<0.001**
Deciduous forest	0.001	0.002	0.272	0.785
Mixed forest	−0.001	0.003	−0.215	0.830
Farmland	−0.001	0.002	−0.506	0.613
**Grassland**	**0.005**	**0.003**	**1.880**	**0.060**
**Urban**	**−0.013**	**0.004**	**−3.234**	**<0.001**
Other	0.003	0.003	0.739	0.460
Land use richness	−0.031	0.053	−0.582	0.561
**Edge density**	**−8.190**	**2.489**	**−3.291**	**<0.001**

**Table 7:** GLM model results accounting for species richness of five *Columbidae* species concerning different land use types, land use richness, and edge density in 2016. The included species are feral pigeon (*Columba livia forma domestica*), stock dove (*Columba oenas*), wood pigeon (*Columba palumbus*), Eurasian collared dove (*Streptopelia decaocto*), and European turtle dove (*Streptopelia turtur*). Abbreviations: SE—standard error. Significant variables are highlighted in bold.

	Estimate	SE	z-Value	*p*-Value
(Intercept)	**0.17**	**0.055**	**3.198**	**0.001**
Deciduous forest	<0.001	0.001	0.149	0.881
Mixed forest	<0.001	0.001	0.011	0.991
Coniferous forest	**−0.001**	**0.001**	**−1.702**	**0.089**
Grassland	−0.001	0.001	−0.993	0.320
Urban	**0.002**	**0.001**	**3.131**	**0.002**
Other	<0.001	0.001	−0.224	0.822
Land use richness	**−0.041**	**0.014**	**−2.995**	**0.003**
Edge density	**1.232**	**0.382**	**3.225**	**0.001**

## Data Availability

The data presented in this study are available on request from the corresponding author.
